# Prevalence of mental disorders among young males in Swedish compulsory residential care

**DOI:** 10.3389/fpsyt.2025.1657469

**Published:** 2025-08-18

**Authors:** Camilla Jalling, Marianne Kristiansson

**Affiliations:** ^1^ Department of Clinical Neuroscience at Karolinska Institutet, Stockholm, Sweden; ^2^ The Swedish National Board of Institutional Care, Solna, Sweden

**Keywords:** compulsory residential care, mental disorder, psychiatric comorbidity, youth & adolescence, juvenile detention, ADHD (attention deficit and hyperactivity disorder), conduct disorder (CD)

## Abstract

**Introduction:**

This study sought to examine the prevalence of diagnosed and undiagnosed psychiatric disorders, comorbidity, and acute psychiatric conditions among young males in compulsory residential care due to criminality, substance misuse, or other socially destructive behavior pattern.

**Methods:**

An online cross-sectional survey measured diagnosed and undiagnosed mental disorders listed in DSM-5, and other psychiatric conditions among children and youth admitted to the Swedish National Board of Institutional Care. Psychologists at the residential homes answered one questionnaire per young male participant by retrieving information from records and other relevant files.

**Results:**

Data for 183 residing young males were analyzed. The prevalence of diagnosed mental disorders was high (77%), and psychiatric comorbidity was present among 46%. ADHD was the most common diagnosis (57%), followed by SUD (20%), CD (14%), ASD (14%), and PTSD (12%). Furthermore, a large proportion of the young males had displayed clinical symptoms without being diagnosed, and 10% had been in a state of acute psychiatric distress.

**Discussion:**

Several findings in this study align with previous research, such as the prevalence of diagnosed conditions and co-morbidities. Some results did diverge, however, and ADHD and SUD were found to have higher prevalences in our study, while CD was considerably lower compared to previous findings. It remains unclear why CD prevalence was low, but it may have to do with the high prevalence of ADHD, and a general reluctance to assess for CD. In Sweden, where this study was carried out, both clinical and general awareness of ADHD has increased over the last two decades, alongside revised diagnostic criteria in DSM-5, which may have improved identification, and assessment, and led to an increase in diagnoses. It may also, in part, have led to clinicians prioritizing assessments of ADHD over CD.

## 1 Introduction

In Sweden, approximately 26,500 of a total of 2 million children and adolescents (1%), are placed in out-of-home care due to inadequate support from current caregivers regarding physical, mental, or social development (1) (psychiatric compulsory care excluded). Most placements in residential care are on a voluntary basis (around 70%) and carried out with support from the Social Services Act (2), while the remaining third are involuntarily according to the Care of Young Persons Act (YPA) (3, 4). The most common form of placement according to YPA is in family homes (70%), and the second (23%) is placement in homes for care or housing (HVB) (6100 individuals annually; 58% boys and 42% girls). In 2022, little over 1100 (18%) of children and adolescents, up to age 21 (of whom, approximately 30% were <15 years old), who were placed in HVB homes were placed in compulsory residential care at the Swedish National Board of Institutional Care (N-BIC) (5).

N-BIC operates a special form of HVB, in accordance with the YPA (1990:52) and provides individually tailored compulsory residential care for children and adolescents who are deemed by social services, and decreed by the Administrative Court, to require close supervision. What distinguishes N-BIC homes from other HVB homes is, for instance, that the staff, under certain conditions, can “use coercive measures, so-called special powers” (1), and that the care homes are subject to high security, with many lockable units. The foundation of N-BIC care is to create stability, establish daily routines, and enhance functional everyday life. N-BIC does not have a mandatory healthcare responsibility; however, it offers clinical assessments, care, and medical drug management. N-BIC is required to cooperate with specialist physicians through collaboration with regional healthcare and to provide access to psychological expertise. N-BIC’s mission is therefore distinct from both psychiatric and standard healthcare services. Consequently, N-BIC relies on collaboration with other healthcare providers for clients to receive necessary medical care.

The criteria for placement in compulsory residential care under YPA are that the children or adolescents themselves have put their health, or their social or psychological development at significant risk of harm due to substance abuse, criminal activity, or other socially destructive behavior. For boys, the primary reasons for placement are criminal activity and/or substance abuse—either individually or in combination (6). When concerns are raised—by, for example, the school, the family, or through the social services’ prior knowledge of the child or family—social services may apply to the administrative court for a decision regarding placement in compulsory residential care.

Also, adolescents 15–18 years of age who have committed severe offenses and been sentenced by criminal court to secure youth care, are placed at specialized secure homes run by N-BIC. Secure care is regulated under the Secure Youth Care Act (7). Most convicted youth are young males: only around 3% of SYCA placements are designated for young woman. Penalties under SYCA are enforced in special secure treatment units and can continue until age 21.

To sum, both children and adolescents with antisocial behaviors and psychosocial dysfunction, and youth convicted for severe criminal acts reside at N-BIC’s youth homes.

Globally, around 8% of children, and 14% of adolescents, live with at least one diagnosed mental disorder (8). In Europe, the annual Lancet Global Burden of Disease Study (GBD) found in a review of 538 individual studies from 31 European countries an increase of mental disorders over the course of the last three decades, and the current prevalence among 10–19 years old children and adolescents is 16%–17% (9). Additionally, results from a Swedish register-based study of 1.4 million adolescents aged 15–20 years old show that 17% had at least one diagnosed mental disorder (data from primary care not included) (10). Mental disorders among convicted and incarcerated young people have, however, repeatedly been shown to be higher in prevalence. For instance, in a recent systematic review it was pointed out that mental disorders—i.e., psychosis, conduct disorder (CD), Attention-Deficit/Hyperactivity Disorder (ADHD), depression, and Post-Traumatic Stress Disorder (PTSD)—were considerably more common among incarcerated adolescents than among the normal population. Among young males, CD was the most commonly diagnosed disorder (at 62%), followed by ADHD (17%), and major depression (10%) (11). The researchers note that, compared to a previous systematic review from 2008, ADHD and CD rates were higher among the incarcerated young males, while no similar increase was observed in the general adolescent population (11, 12).

Psychiatric conditions and disorders among children and adolescents admitted or sentenced to N-BIC to receive care in accordance with YPA and SYCA have been studied previously by Ståhlberg and colleagues (13). Children and adolescents placed at a selection of N-BIC residential homes were assessed for mental health following various protocols, including DSM-IV criteria for several mental disorders. The results show that the majority fulfilled the criteria for at least one mental disorder (73%, not counting CD—being the main reasons for placement at N-BIC). DSM-IV criteria for CD were fulfilled for 77% of the adolescents, substance use disorder for 55%, ADHD for 48%, autism spectrum disorder for 17%, and intellectual disability for 10% (13). Furthermore, in 2018, the Swedish National Board of Health and Welfare mapped psychiatric care needs among children and adolescents placed at N-BIC in accordance with YPA and found high prevalences of diagnosed mental disorders (71%) and psychiatric comorbidities (45%). The most commonly diagnosed disorders were ADHD (44%), followed by SUD (23%), PTSD (16%), CD (16%), and anxiety (10%) (14).

In addition to the above presented research findings, updated data on psychiatric care needs are crucial for organizations that administrate care and treatment that addresses antisocial development among both convicted and pre-convicted young males. Besides this—and perhaps just as important—is the urgent need for knowledge regarding psychiatric conditions in young people who are at risk of becoming involved in, or who are already involved in, serious criminal activities. Sweden’s current situation, in which criminal gangs are engaging in gun violence—gangs that exploit children in their early teens or even younger, urging them to commit serious and frequently violent crimes—calls for additional research perspectives beyond those in the social sciences.

Up-to-date data on psychiatric conditions among adolescents sentenced for severe offenses, as well as those exhibiting antisocial behavioral development, will support organizational planning, intervention development, and the implementation of appropriate measures to prevent relapse into antisocial behavior and criminality.

The current study is part of a research project on mental healthcare needs among children, adolescents, and adults with substance use disorder in compulsory residential care in Sweden, ethically approved by The Swedish Ethical Review Authority (ref nr 2022-00910-01). The present study presents the results from young males aged 16–21 placed in compulsory residential care at N-BIC to receive care in accordance with YPA or SYCA.

### 1.1 Aims and research questions

The aim was to study mental disorders and psychiatric conditions among young males at N-BIC. More specifically, the aim was to study the prevalence of mental disorders and psychiatric comorbidity, how common acute psychiatric conditions were and receiving interventions due to such conditions, and whether there were any differences between young males under the care of YPA or SYCA in regard to the prevalence of mental disorders, psychiatric comorbidity, or acute conditions.

## 2 Materials and methods

The current study is part of a large research project using a cross-sectional study design aiming to include children and adolescents, and adults with substance use disorder, who were admitted to N-BIC on May 2, 2022. The methodology followed a similar approach to that of the Swedish National Board of Health and Welfare, which mapped children and adolescents admitted to N-BIC under the YPA (14), excluding individuals admitted in accordance to SYCA.

The response rate for males was 53% and consisted of data for 219 males of the total admitted 412 males. The females who were admitted at the time were not included in this paper; instead, this partial study uses data from a sub-sample comprised of young males aged 16–21 years admitted to N-BIC.

Using REDCap software, data collection was conducted by psychologists at the residential homes, who filled out one web-based questionnaire per young male participant. Information was retrieved from each young male’s records and other relevant registered files. In total, data for 183 young males was included, and missing data was due to opt-out, un-answered questionnaires, and absent young males. No young males actively participated in data collection themselves

### 2.1 Procedure

The psychologists at N-BIC informed young males about the study and its implementation at regular ward meetings and via posters at each ward. Both the oral and written information made it clear that they could opt out of the study so that their data would not be collected. Research participants whom the psychologist assessed as having special needs were informed about the study through individual conversations. If the young male was deemed unable to speak for themselves, the guardian or legal representative was informed about the study.

The questionnaires were completed by the psychologists, who retrieved information from health and medical records in TakeCare and from the N-BIC’s client administrative journal system (KAJ). No data regarding names, personal identification numbers, addresses, case numbers, or names of wards or institutions were requested, ensuring the collected data remained pseudonymized. Additionally, no information was requested about the psychologist who filled out the web surveys. The data collection period was just over three months long and ended August 30, 2022.

#### 2.1.1 Questionnaire

The questionnaire was based on a previous survey on YPA children that the Swedish National Board of Health and Welfare conducted in 2018 (14), with a minor revision.

The present questionnaire focused on diagnosed mental disorders and psychiatric clinical symptoms, in accordance with the DSM-5 (Diagnostic and Statistical Manual of Mental Disorders, 5th ed. [15]) and/or ICD-10 (International Statistical Classification of Diseases and Related Health Problems—Tenth Revision [16]). The diagnoses were: Attention-Deficit/Hyperactivity Disorder (ADHD), Anxiety Disorders, Antisocial Personality Disorder (APD), Autism Spectrum Disorder (ASD), Bipolar Disorder; Borderline Personality Disorder (BPD), Conduct Disorder (CD), Depressive Disorder (DP), Eating Disorders, Intellectual Disability, Obsessive Compulsory Disorder (OCD), Opposition Defiant Disorder (ODD), Post-Traumatic Stress Disorder (PTSD), Psychotic Disorders, and Substance Use Disorder (SUD). On each of these 15 listed disorders, psychologists reported the presence of any diagnoses and when the young male was diagnosed (response alternatives “yes: during 2020–2022”, “yes: 2019 or before”, “yes: no information on when”, or “no”). Questions were asked as to whether undiagnosed young males had displayed symptoms of each of the listed mental disorders, and if so, to what degree. To rate the severity of any symptoms, information was retrieved from medical records, notes, or other documentation, and on the psychologist’s personal perception (response alternatives: severe, high, some; none). Furthermore, the questionnaire covered acute psychiatric conditions. Acute psychiatric conditions were defined as a condition in which the young male participant had needed immediate care during the past six months, due to suicidal behavior, suicide attempts, self-harm, or because of an eating disorder, panic attack, dissociative episode, psychotic episode, or ‘other’ (where psychologists were asked to report what prompted the need for immediate care). Psychologists were also asked to report whether the young male had committed any suicide attempts during their stay at N-BIC, and if so, how many times (response options: ‘none’, ‘one time’, ‘2–5 times’, ‘more than 5 times’). Lastly, psychologists reported whether the young male had received any of the following acute interventions during their stay at N-BIC: suicide risk assessment, periodic supervision, constant supervision, or ‘other’ (where psychologists were asked to report which acute interventions had been performed).

### 2.2 Data

Background data were presented with proportions. Data on diagnosed mental disorders were recoded into binary variables (‘yes’ or ‘no’). Also, data on symptoms of any disorder were recoded to binary variables, by combining the options ‘severe’ and ‘high’ into 1= ‘clinical symptoms’, indicating that the young male had displayed symptoms on a clinical level. All other options were coded as 0, except for when the option ‘insufficient available information’ was checked. These instances were omitted from the analyses on psychiatric symptoms. Only young males without a diagnosis of any disorder were included in the analyses on the same clinical symptom, which is why *n* differs in each analysis. Tests for significant differences between young males remanded to the care of YPA and SYCA were conducted with Pearson’s Chi-square tests.

## 3 Results

**Figure 1:**
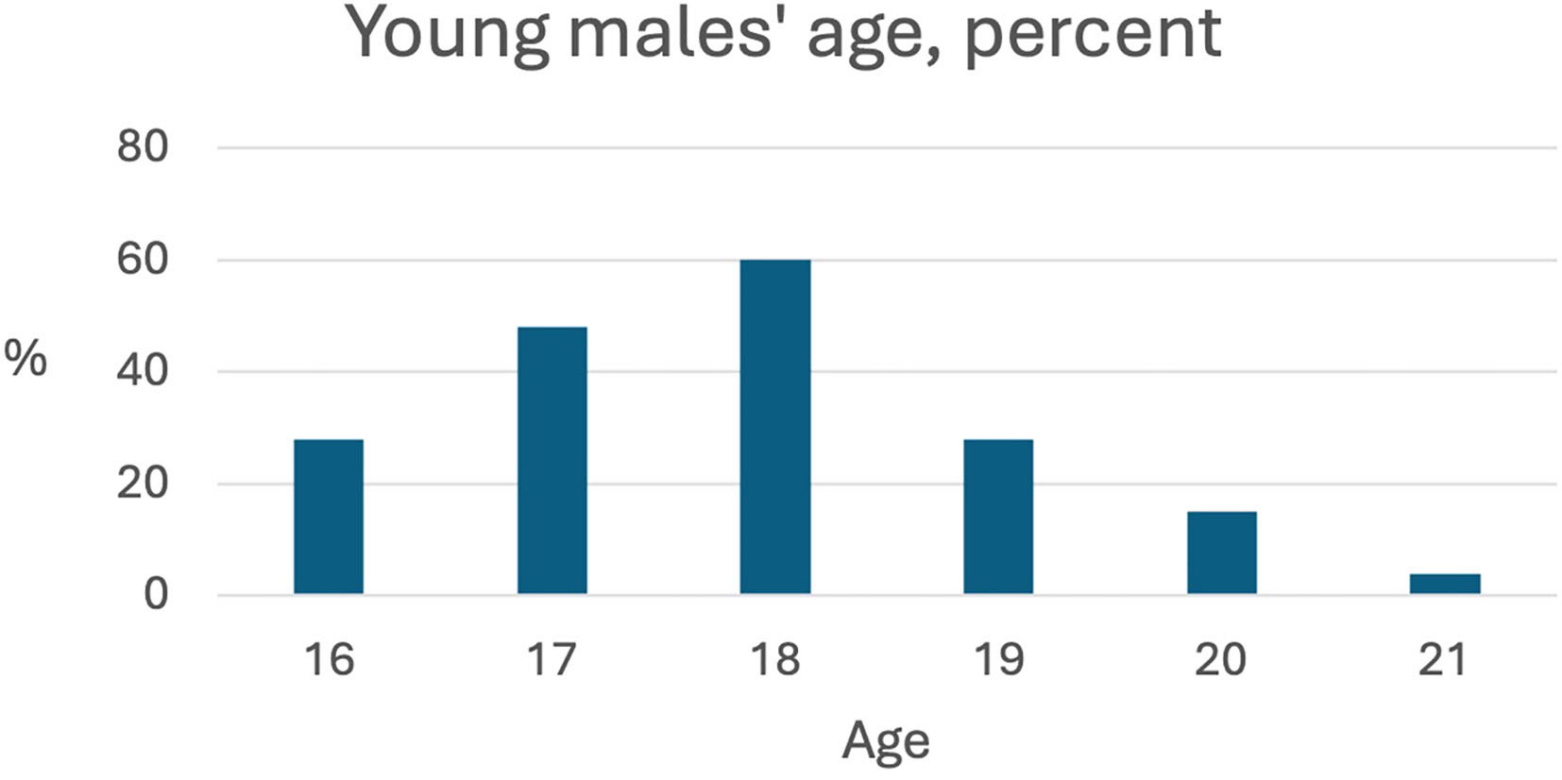
Distribution of the young male participants’ age, by percentage.

Data for 183 young males were included in the analyses (148 YPA males and 35 SYCA males), aged between 16 and 21 years (*M* =17.81, *SD* =1.23). **Figure 1** shows the age distribution.

### 3.1 Diagnosed mental disorders

Just below 77% of the young males had at least one diagnosed mental disorder. Psychiatric comorbidity was common among the young males (*M* =1.62, *SD* =1.39), and almost half of them (46%) had two or more diagnosed mental disorders. Approximately one of four (25%) young males were diagnosed with three or more mental disorders (**Table 1**). There were no significant differences between the YPA and SYCA participants.

**Table 1:** Proportion of young males with number of diagnosed mental disorders (%).

Number of diagnoses	Proportion
0	23
1	31
2	21
3	14
4	8
5	(-)
6	(-)

(-), if less than five individuals.

The most commonly diagnosed mental disorder was ADHD (57%), followed by SUD (20%) AUD (14%), CD (14%) and PTSD (12%), see **Table 2**. Around 28% of the young males without a diagnosis for SUD displayed clinical symptoms of the condition. Also, 20% had displayed clinical symptoms of CD, 12% for ODD, and 10% for PTSD, without having been diagnosed with the specific condition. Comparisons of YPA and SYCA males showed no significant differences in diagnosed conditions or undiagnosed conditions (i.e., in displayed clinical symptoms).

**Table 2:** Proportions of diagnosed mental disorders and displayed clinical symptoms of undiagnosed disorders (%).

Mental disorders	Diagnoses	Clinical symptoms
ADHD	57	9 (n =79)
Anxiety Disorders	8	9 (n =168)
Antisocial Personality Disorder (APD)	(-)	8 (n =179)
Autism Spectrum Disorder (ASD)	14	(-) (n =158)
Bipolar Disorder	–	–
Borderline Personality Disorder (BPD)	–	(-) (n =183)
Conduct Disorder (CD)	14	20 (n =158)
Depressive Disorders	9	4 (n =167)
Eating Disorders	–	–
Intellectual Disability	4	(-) (n =175)
Obsessive Compulsory Disorder (OCD)	4	(-) (n =172)
Opposition Defiant Disorder (ODD)	8	12 (n =169)
Post-Traumatic Stress Disorder (PTSD)	12	10 (n =161)
Psychotic Disorders	(-)	–
Substance Use Disorder (SUD)	20	28 (n =146)

(-), if less than five individuals. -, none.

Almost 22% were scheduled for treatment within child and youth psychiatric care, which was more common among young males admitted in accordance with YPA (25%) compared to SYCA (12%) (just above the significance level of .5; i.e., [χ^2^ (df 2) = 5.57, *p* =.062]. Almost 9% were unable to answer the question.

### 3.2 Acute psychiatric conditions

Just above 8% of the young males had been in a state of acute psychiatric condition during the last six months. There was a significant difference between young males admitted in accordance with YPA and SYCA (10% of YPA, none of SYCA [χ^2^ (df 1) =3.87, *p* =.049]). Questions were asked regarding whether, and if so, how many times the young males had been in a state of the following acute psychiatric conditions: suicidal behavior (attempts excluded), suicidal attempt, self-harm, eating disorder, psychosis, panic attack, dissociative state, or any other acute condition. For the listed conditions, fewer than five young males had been in an acute state during the last six months (with the exception of no young males with a condition related to eating disorders or a dissociative state). According to these results, fewer than five of the participants had received immediate care due to any of the acute psychiatric conditions listed above.

## 4 Discussion

In this study, we found a high prevalence of diagnosed mental disorders among the young male participants aged 16–21 years in residential compulsory care in accordance with YPA or SYCA, at the Swedish National Board of Institutional Care (N-BIC). Mental disorders were found in 77% of the young males, and 46% presented psychiatric comorbidity, with two diagnosed disorders or more. Every fourth young male had three diagnosed disorders or more (25%). The most prevalent diagnoses were ADHD (57%), SUD (20%), CD (14%), and AUD (14%). Also, a large proportion of the young males had displayed clinically relevant symptoms of different undiagnosed psychiatric conditions, and 20% had displayed clinical levels of symptoms of CD, and 28% of SUD, without having been diagnosed with the condition. One in ten young males admitted in accordance with YPA had been in an acute state of psychiatric distress.

### 4.1 Prevalence of ADHD, conduct disorder, and substance use disorder

Many of our findings were consistent with previous research; however, some findings diverged. Diagnosed ADHD was found to be far more common in the present study (57%), compared to rates below 20% reported in international meta-analyses (11, 12, 17–19). Globally, the prevalence of ADHD in the general population—encompassing both diagnosed and undiagnosed cases—has remained stable at just under 6% over the past three decades (20, 21). In both Swedish and American large general population studies of diagnosed ADHD, however, an upward trend has been identified (22–24). Beaudry et al. (11) discuss the increase in diagnosed ADHD among juvenile detainees in comparison to the findings reported by Fazel et al. (12) and suggest that it may be a result of clinical awareness leading to both increased and improved identification and assessment procedures (11). In addition, other research suggests that the revised diagnostic criteria in DSM-5 compared to DSM-IV may have led to at least a part of the increase in diagnosed ADHD, rather than an actual increase in an overall prevalence of ADHD (23, 25, 26). The high prevalence of diagnosed ADHD in Sweden—including young males at N-BIC—may be explained by a larger increase in general and clinical awareness of ADHD over the last decade, and improved routines for reporting diagnoses in national registers, as suggested by the Swedish National Board of Health and Welfare in a report from 2014 (26). Additionally, a Swedish healthcare initiative known as “ADHD Fast Track”, which introduced a shortened and streamlined diagnostic process, may have contributed to the observed increase in prevalence.

Our results also differed from previous research regarding diagnosed conduct disorder (CD). We found that 14% of the young males had a formal CD diagnosis, and additional 20% had displayed clinical symptoms of undiagnosed CD, while previous research has found considerably higher prevalence rates. For instance, in a Swedish study of young people placed at N-BIC in accordance with YPA and SYCA, 77% met the DSM-IV criteria for a CD diagnosis (13). Despite focusing on the same population—i.e., young people admitted to N-BIC—as the present study, Ståhlberg et al. (13) reported a considerably higher prevalence of CD. Although the studies were conducted 15 years apart (Ståhlberg’s data collection year 2007, the present study year 2022), we assume that the discrepancy is primarily related to differences in methodology. Ståhlberg et al. (13) assessed individuals aged 12 to 19 years for mental health problems using various structured protocols, including DSM-IV criteria for several psychiatric disorders. In contrast, the present study relied on recorded diagnoses retrieved from medical records. A comprehensive, individual assessment of mental disorders, as conducted by Ståhlberg et al. (13), would likely yield a more accurate estimate compared to relying solely on documented diagnoses—on which the current study was based.

Findings from the report of Socialstyrelsen (14) support this interpretation, as they employed a similar methodology and found a CD prevalence rate comparable to that in the present study. However, unlike the Socialstyrelsen’s study, which included children and adolescents placed at N-BIC in accordance with the YPA, the present study focused on young males aged 15 and older, including convicted offenders—a group from which higher CD prevalence might be expected. Also, in Swedish child and youth residential care, including compulsory residential care, the provided care traditionally has a strong focus on social factors, such as school, family, social relations at the ward and elsewhere, and daily routine functioning, why other individual factors such as antisocial development and norm breaking behavior and co-existing mental disorders may not be in primarily focus. This could at least partially influence the reluctance to conduct assessments of CD.

Also, in previous meta-analyses, CD prevalence ranges between 46.4% and 68% among incarcerated young males (11, 12, 17, 18). It remains unclear as to why the discrepancy between our results and previous research is so large. The observed discrepancy may be partly explained by a general Swedish tendency to prioritize the formal assessment of ADHD, rather than CD. For instance, CD is common among individuals with neuropsychiatric disorders, such as ADHD (15), and symptoms may be interpreted as overlapping. Given the earlier suggestion regarding increased clinical awareness and the implementation of the “ADHD fast track” initiative, investigation of ADHD may have been prioritized over CD. Besides that, children with CD have an increased risk of ADHD, making differential diagnostics challenging but imperative (27). Moreover, a CD diagnosis may be perceived as particularly stigmatizing, which could result in reluctance among staff at N-BIC and child and youth psychiatric care to pursue formal assessment. This would imply a psychiatric comorbidity between diagnosed ADHD and undiagnosed CD, which has, however, not been assessed in this study. To sum up, one interpretation of the above reasoning is that CD may be underdiagnosed in the population included in the present study.

Compared to findings of substance use disorder (SUD) in the general European youth population (4%, see the Lancet Global Burden of Disease Study, 9), prevalence in the present study was considerably higher (diagnosed 20%, and undiagnosed 28%). The high prevalence of SUD among incarcerated male youth has been confirmed by previous research (28), as has its link to other psychiatric disorders (29, 30). Risky or binge drinking at an early age and its impact on risk-taking and impulsiveness (31)—and the impact of teenage drinking on brain development and cognitive functioning in regard to learning, attention, memory, and the regulation of affection and impulsivity—implies that early identification, assessment, and treatment is deeply important to be able to prevent harm and the further development of addiction. Among adults in compulsory addiction care, one of the strongest associations between repeated admittances to compulsory addiction care is prior placement in compulsory residential care as child or youth (32, 33). Therefore, among youth in compulsory care, the importance of identification, assessment, and treatment of SUD could not be highlighted enough.

### 4.2 Undiagnosed psychiatric and acute conditions

From a clinical perspective, undiagnosed conditions eventually may become apparent to staff, which places a strain on their resources, as they work to map the clinical picture and meet individuals’ needs. One obvious need is treatment: either through medication, therapy, or other intervention. Also, in settings with demanding residents, it’s important to create safe and predictable environments for both staff and for the residents themselves. The large number of undiagnosed conditions among young males at N-BIC points out the need for prompt examination and diagnostics, when applicable. In this group—i.e., young males who present with antisocial behavior or antisocial behavior development—the need for prompt examination, diagnostics, and treatment is not only necessary for the individual and for the residential organizational, but also an urgent societal need. In Sweden, where this study was conducted, there is a current concerning development involving criminal gangs consisting of children and youth who engage in severe offenses such as shootings and setting off explosions. A considerable number of children and adolescents at N-BIC currently consists of such gang members, which points to the urgent necessity of several targeted individual interventions, including proper treatment for psychiatric conditions.

Our findings also point to the considerable need for psychiatric care among young males in Swedish compulsory residential care. For instance, one in 10 young males had at least one acute condition during the last six months that required immediate care from staff at N-BIC, psychiatric units, or emergency care. This was, however, not the case among young males admitted in accordance with SYCA, highlighting that YPA males had significantly more often suffered from acute psychiatric conditions.

### 4.3 Concluding remarks

To conclude, results from the present Swedish study of young males in compulsory residential care present a massive burden of psychiatric diagnoses. Many of the studied young males engaged in norm-breaking behavior, and it was unexpected that the prevalence of CD was lower compared to many other studies. Research has consistently shown that youth involved in the justice system and institutional care due to criminal behavior exhibit significantly higher rates of CD compared to the general population (10–12, 18, 30, 34). This warrants further research. It is conceivable that there might be subgroups of CD, some with impulsivity and some with callous unemotional traits, which also must be delineated. Research also highlights the heterogeneity of CD, suggesting that a more nuanced understanding could be achieved by applying a comprehensive specifier model that includes Grandiose–Manipulative (GM) and Irresponsible–Impulsive (II) traits, in addition to CU traits (35). It might also be that those individuals suffering from CD, along with callous unemotional traits, may be more involved in instrumental violence. It is beneficial to better understand subgroups of CD to better tailor relevant interventions.

#### 4.3.1 Study limits

This cross-sectional study utilized data retrieved from individual records maintained by N-BIC. The data concerned registered psychiatric diagnoses, which were either based on information provided by social services during the application process for placement at N-BIC, or derived from clinical assessments and diagnostic procedures enacted at N-BIC. It is important to note that psychiatric diagnoses established and recorded within regional healthcare or psychiatry services, within or without the regional healthcare—i.e., those documented in their respective medical record systems—may not be included in this study. This is because N-BIC maintains separate records and is not permitted to integrate or cross-reference data with healthcare or psychiatric registers, or vice versa. In cases where social services lacked information stemming directly from the youth or their family, such information was not included in the application, and, consequently, was not registered in the dataset. This entails that the prevalence of diagnoses in this study is most likely under-estimated.

The study is limited by a response rate of 53%. This rate can be compared to that of a study conducted by the Swedish National Board of Health and Welfare (14), which used a methodology similar to that employed in the present study. Their study included data on children and adolescents admitted to N-BIC in accordance with YPA only—i.e. omitting adolescents placed in accordance with SYCA—and achieved a response rate of 75% (14). Both studies relied entirely on the participating psychologists’ willingness and availability to complete the questionnaires. Given that these were clinical psychologists, often working in understaffed settings, it is likely that clinical duties were prioritized over research participation when our data collection were conducted.

Another notable difference between the two data collections is that the Swedish National Board of Health and Welfare conducted their study as part of a government commission to map the psychiatric care needs of children and adolescents at N-BIC—a type of assignment that is typically prioritized and endorsed by Director-Generals. This may have increased the perceived obligation to fill out questionnaires, thereby contributing to the higher response rate.

I addition, the data collection by the Swedish National Board of Health and Welfare used pen-and-paper method, while the present study was a web-based questionnaire. According to a recent systematic review on possible differences between web surveys and alternative data collection methods in epidemiological and public health studies, found in the 19 included studies that response rate from web-based data collection was in general 12.9 percentage points lower than with alternative methods (web survey’s response rate 40.5%, alternative methods 56.3%, when also randomized controlled trials were included) (36). The authors conclude that despite lower response rate, web surveys have advantages over alternative methods such as more efficient data consistency, and better data quality (36). Despite that systematically lower respons rate in web surveys is common, and is expected to not negatively affect data quality, and that results were comparable with the study of the Swedish National Board of Health and Welfare, the present study’s results should be interpreted with caution.

With regards to response rate, it is possible that some of the statistical comparisons between SYCA and YPA in the present study might have reached significance if the sample size, including the response rate, had been larger. Finally, the relatively low response rate may affect the generalizability of the findings.

Another identified limitation of the study is the estimation of clinical symptoms of undiagnosed psychiatric conditions. To answer these questions, the psychologist was asked to search for information in the records, in other relevant documents, or to base the answer on his/her own experiences with the relevant individual. Therefore, data on displayed clinical symptoms are based on individual conception using information from the staffs’ or psychologist’s notes or their own experience, and not on clinical assessments. At the best, the data presented in this study may indicate that some psychiatric conditions are under-diagnosed, and that staff need to be attentive to symptoms, and upon suspicion conduct clinical assessments and diagnostic procedures, since a diagnosis helps in planning treatment.

## Data Availability

The datasets presented in this article are not readily available because they constitute working material in ongoing studies. Requests to access these datasets should be directed to the corresponding author.
